# ﻿*Porroglossum hildeae* sp. nov. (Orchidaceae), a new species from the threatened cloud forests of northwestern Ecuador

**DOI:** 10.3897/phytokeys.263.159826

**Published:** 2025-09-24

**Authors:** Marco F. Monteros, Eugenio Restrepo, Gabriel A. Iturralde, Marco M. Jiménez, Luis E. Baquero

**Affiliations:** 1 Fundación EcoMinga, 270 12 de noviembre and Luis A Martínez, Baños, Tungurahua, Ecuador Fundación EcoMinga Baños Ecuador; 2 Reserva: The Youth Land Trust, Washington, D.C., USA Grupo Científico Calaway Dodson: Investigación y Conservación de Orquídeas del Ecuador Quito Ecuador; 3 Grupo Científico Calaway Dodson: Investigación y Conservación de Orquídeas del Ecuador, Quito, 170510, Pichincha, Ecuador Reserva: The Youth Land Trus Washington United States of America; 4 Instituto Nacional de Biodiversidad (INABIO), Rumipamba 341 y Av. De los Shyris, Quito, Ecuador Instituto Nacional de Biodiversidad (INABIO) Quito Ecuador; 5 Grupo de Investigación Schultes, Fundación Ecotonos, Carrera 72 #13ª-56, Cali, Colombia Grupo de Investigación Schultes, Fundación Ecotonos Cali Colombia; 6 Grupo de Investigación en Biodiversidad, Medio Ambiente y Salud (BIOMAS), Carrera de Ingeniería en Agroindustria, Facultad de Ingenierías y Ciencias Aplicadas, Universidad de Las Américas, UDLA, Vía a Nayón, Quito 170124, Ecuador Universidad de Las Américas Quito Ecuador

**Keywords:** Conservation, Cordillera del Toisán, epiphyte, Intag Valley, IUCN Red List, Río Manduriacu Reserve

## Abstract

A new species of *Porroglossum* (Orchidaceae) is described and illustrated from the cloud forests of northwestern Ecuador. *Porroglossum hildeae***sp. nov.** is morphologically similar to *P. josei* Luer, but can be distinguished by several features: it has narrowly obovate leaves reaching up to 9 cm in length (vs. elliptical, 4.5 cm), a longer dorsal sepaline tail measuring 6 mm (vs. 1.5 mm), oblong-ovate petals with one acute angle at the upper margin (vs. ovate with the upper and lower margins acutely angled below the middle), and the lip obtrullate, attenuate at the base, and acute at the apex (vs. obovate with the apex obtuse and abruptly acuminate). It grows epiphytically at approximately 1,600 m elevation in a cloud forest ecosystem that is increasingly threatened by deforestation, land-use change, and mining activities. Due to its restricted distribution and the ongoing degradation of its habitat, we recommend classifying it as Critically Endangered according to IUCN Red List criteria.

## ﻿Introduction

The genus *Porroglossum* Schlechter is a Neotropical group of orchids comprising approximately 56 accepted taxa distributed along the Andean cloud forests from Venezuela to Bolivia (Luer 1987; POWO 2024). Its center of diversity lies in Ecuador where over 30 species are currently known, many of which are endemic (Baquero and Iturralde 2017; Baquero et al. 2020). Since the last comprehensive taxonomic revision of the genus (Luer 1987), 36 additional species have been described, significantly expanding the taxonomic knowledge of the group (IPNI 2025). Phylogenetic studies based on molecular evidence consistently place *Porroglossum* within the *Masdevallia* alliance, alongside *Dracula*, *Trisetella*, and *Diodonopsis* (Karremans 2016). Unlike many genera in the subtribe, *Porroglossum* is a well-supported monophyletic group, characterized by a distinctive and highly specialized floral mechanism; a mobile, touch-sensitive lip that acts as a trap to facilitate pollination, primarily by drosophilid flies (Luer 1987; Karremans and Díaz-Morales 2019).

Ecuador is known for its rich biodiversity and is home to an astonishingly rich orchid flora. Approximately one-third of the total endemic vascular plant species of Ecuador are orchids (e.g. Endara et al. 2009; León-Yánez et al. 2011). The northwestern region of Ecuador, particularly the Cordillera del Toisán, exhibits significant orchid diversity, as shown by the recently published new species of Orchidaceae: *Lepanthes inesmanzanoae* M.F.Monteros & E.Restrepo, *Masdevallia purocafeana* M.F. Monteros & Baquero, *Platystele cedriensis* Baquero & G.Verkovitch and *P. decouxii* Baquero & G.Verkovitch (Baquero and Galarza-Verkovitch 2019; Monteros et al. 2022; Monteros et al. 2023).

In this region, deforestation and the expansion of agricultural and livestock frontiers have caused the loss of native vegetation cover (Roy et al. 2018). In this sense, the role of conservation areas such as the Cotacachi Cayapas National Park (PNCC), the Intag Toisán Municipal Conservation and Sustainable Use Area (ACUSMIT), protected forests, water protection areas, community reserves, and private conservation efforts such as the Río Manduriacu Reserve managed by the EcoMinga Foundation, contribute to the conservation of diverse ecosystems that include both endemic and threatened species (Torres-Jiménez et al. 2024; KBA 2025). During field monitoring carried out in the Río Manduriacu Reserve between 2019 and 2024, an undocumented species of the genus *Porroglossum* was recorded, which we describe and illustrate herein, along with providing information on its habitat, distribution, and proposed conservation status.

## ﻿Materials and methods

Fertile material was collected during scientific expeditions conducted between 2021 and 2024 to document the orchid diversity of the Río Manduriacu Reserve. Vegetative parts were dried and prepared as herbarium specimens while flowers were preserved in a solution of 70% ethanol and 1% glycerol. The dried specimens were deposited at the National Herbarium of Ecuador (QCNE). Floral and vegetative structures were dissected and photographed using a Canon EOS T6 camera equipped with a Canon EF-S 35/2.8 macro lens. Composite plates and digital line drawings were prepared from images of the holotype and paratype specimens using Adobe Photoshop® 2019.

Collected specimens and digital images were compared with morphologically similar species using specialized literature focused on the genus *Porroglossum* (Luer 1987, 1988, 1989, 1991, 1994, 1995, 1998, 2006, 2010, 2011; Merino et al. 2010; Luer and Thoerle 2012, 2013; Kolanowska and Szlachetko 2013; McDaniel et al. 2015; Baquero and Iturralde 2017; Baquero et al. 2020; Parra-Sánchez et al. 2024). The species description followed the botanical terminology of Beentje (2012), Lindley (1951), and the inflorescence terminology proposed by Rojas-Alvarado and Karremans (2024). The conservation status and Area of Occupancy (AOO) of the newly described species were assessed following IUCN Red List criteria, using the GeoCAT tool (Bachman et al. 2011; IUCN 2022).

Distribution and threat maps were generated using ArcMap v.10. Verified occurrence records of the species were compiled and integrated with thematic layers of vegetation cover, conservation areas, mining concessions, and hydrographic networks (ARCOM 2018; Instituto Geográfico Militar 2020; Ministerio de Ambiente, Agua y Transición Ecológica 2025). Spatial analyses were performed to delineate the AOO and to identify potential threats within the distribution range. All cartographic bases and geographic information layers were obtained from the official information bases of Ecuador.

## ﻿Taxonomic treatment

### ﻿ Porroglossum hildeae

Taxon classificationPlantaeAsparagalesOrchidaceae

M.F.Monteros, E.Restrepo & Baquero sp. nov.

301852B2-0485-50FD-A867-56BBDC304A50

urn:lsid:ipni.org:names:77369660-1

Figs 1, 2, 3

#### Holotype.

Ecuador • Imbabura: Reserva Río Manduriacu, 11 July 2021 (coordinates omitted for conservation reasons; detailed data on the herbarium Type specimen), *Marco F. Monteros, MFM212* (QCNE!).

**Figure 1.**
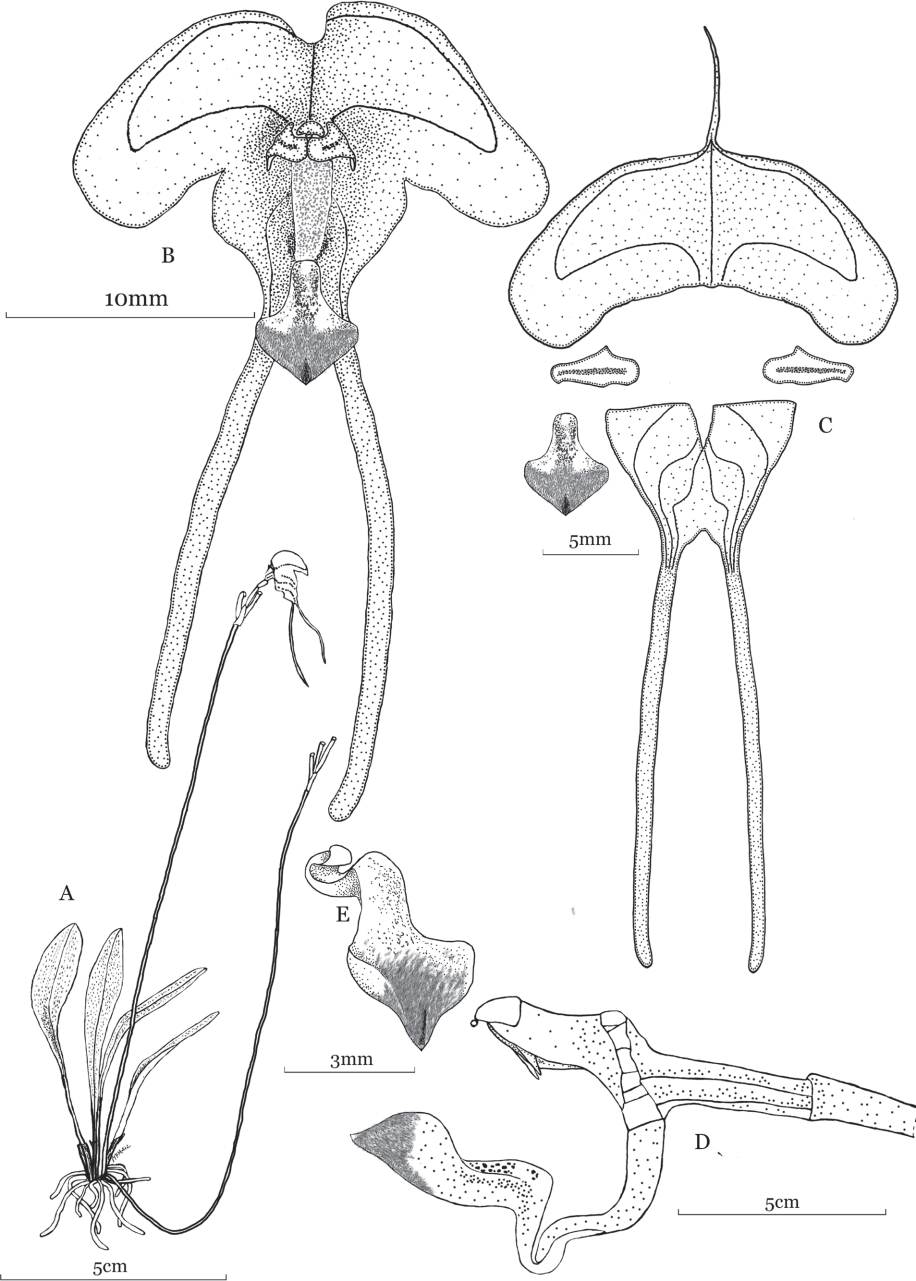
Illustration of *Porroglossum hildeae* M.F.Monteros, E.Restrepo & Baquero, sp. nov. A. Habit; B. Flower, frontal view; C. Dissected flower; D. Ovary, column and partially closed lip; E. Lip ¾ view. Drawn by M.F. Monteros from the plant that served as the type (Monteros 212, QCNE).

#### Diagnosis.

Most similar to *P. josei* Luer but distinguished by the longer, narrowly obovate leaves 8.0–9.0 cm long (vs. shorter, elliptical, 4.5 cm long), tail length of the dorsal sepal 6 mm long (vs. 1.5 mm long), longer lateral sepaline tails 19–20 mm long (vs. ca. 14 mm), petals oblong-ovate with one acute angle near the middle of the upper margin (vs. ovate with two acute angles below the middle), obtrullate blade of the lip with an attenuate base (vs. obovate) and apex of the lip acute (vs. obtuse and abruptly acuminate).

**Figure 2.**
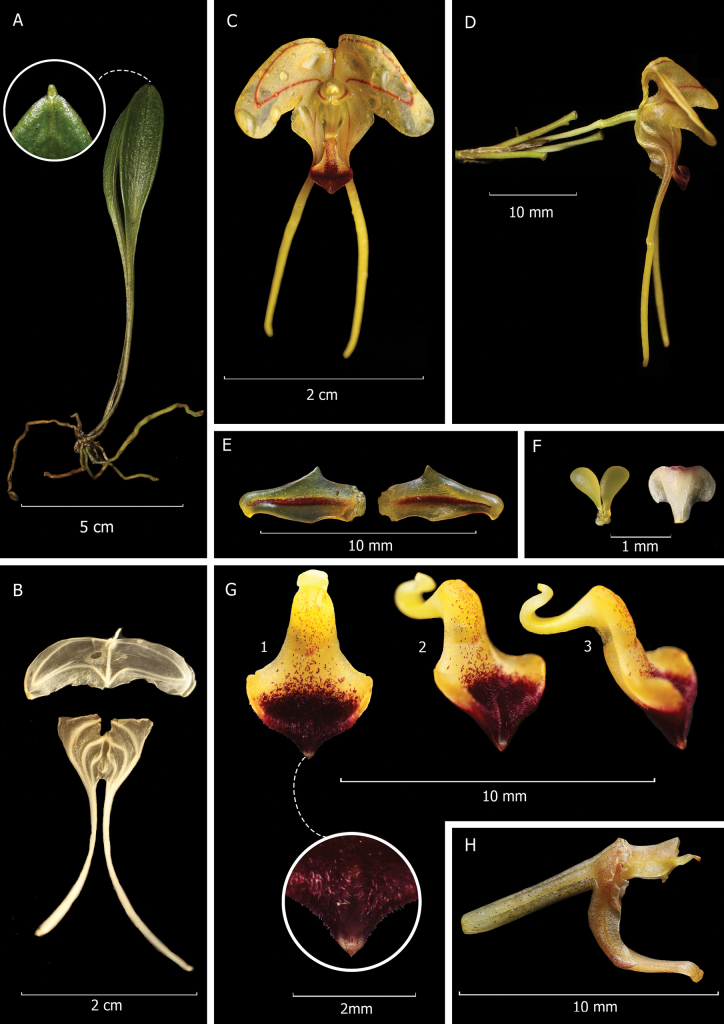
Composite plate of *Porroglossum hildeae* M.F.Monteros, E.Restrepo & Baquero. A. Plant; B. Frontal view of dissected perianth (holotype: flower preserved in alcohol); C. Frontal view of flower (holotype); D. Lateral view of flower; E. Petals (holotype); F. Anther cap, and pollinarium; G1. Lip frontal view (paratype); G2. Lip ¾ view (paratype); G3. Lip lateral view (paratype) H. Ovary and column and column foot. Photographs by M. F. Monteros from holotype (Monteros 212, QCNE) and paratype (Monteros 312, QCNE).

#### Description.

***Plant*** epiphytic, caespitose, up to 20.0 cm tall including the inflorescence. ***Roots*** slender, ca. 1.0 mm in diameter. ***Ramicauls*** erect, slender, ca. 2.0 cm long, enclosed by 2–3 tubular sheaths. ***Leaf*** erect, coriaceous, verrucose, long-petiolate, 8.8–9.2 cm including the petiole 3.3–3.7 cm long; the blade 4.6–5.7 × 0.9–1.2 cm, narrowly obovate, the apex obtuse, mucronate; the base narrowed into a slender, conduplicate petiole. ***Inflorescence*** borne at the apex of the ramicaul, with few-flowered, successive, congested coflorescences, one developing at a time, erect to suberect, with slowly successive flowers, 1 opened at a time, up to 27.0 cm long, including the slender, glabrous pseudopeduncle 17.0–24.5 cm long, with 3–4 internodes, with a few widely spaced thin translucent bracts. ***Floral bracts*** tubular, imbricating, 7.0–10.0 mm long; pedicel 10.0–11.6 mm long. ***Ovary*** glabrous, sulcate, minutely foveolate, 4.0 mm long. ***Flowers*** resupinate with a strong vanilla and clove fragrance in the early evening. ***Sepals*** bright orange-yellow, hyaline, commonly with red veins. ***Dorsal sepal*** reniform, 3-veined, 5.0–7.0 × 17.0–20.0 mm when expanded, connate to the lateral sepals for 3 mm to form a gaping cup, the apex acutely reflexed, abruptly contracted into a tail ca. 6.0 mm long. ***Lateral sepals*** ovate, oblique, 3-veined, 6.0–7.0 × 9.0–10.0 mm, connate 5 mm to form an acute mentum below the column-foot; the apex oblique, acute, contracted into a slender tail, 19.0–20.0 mm long. ***Petals*** bright yellow with an orange-colored midvein, oblong-ovate, 4 × 2 mm, narrowed to the slightly, clawed, rounded apex, the upper margin with an acute angle near the middle. ***Lip*** bright orange-yellow, with dark, brown-red that extends from the apex toward the apical third of the callus gradually degrading into very small spots at the base of the callus, the blade shortly pubescent toward the apex, obtrullate, 7.4 × 4.1 mm, sensitive and actively motile, the thickened callus with a shortly elevated keel running longitudinally towards the base; the apex attenuate, acute, with a sulcus running 1.5 mm from the apex to the center of the blade; the base deflexed and hinged to the free apex of the column-foot by a strap-like claw. ***Column*** greenish-cream, stout, semi-terete, 3 mm long, with tooth-like processes near the stigma, the column-foot slender, curved, 6 mm long. ***Pollinia*** 2, greenish-yellow, obovate, each with a glandular caudicle, 1 mm long. ***Fruits and seeds*** not seen.

**Figure 3.**
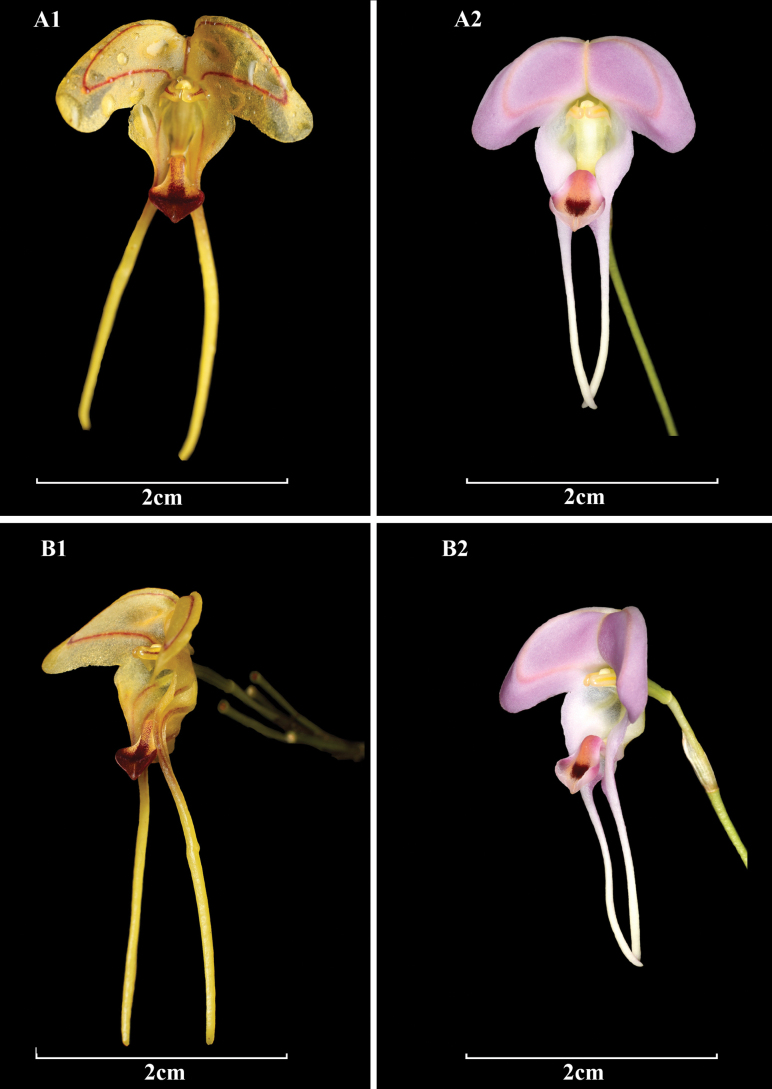
Comparison of A. *Porroglossum hildeae* M.F.Monteros, E.Restrepo & Baquero and B. *P. josei* Luer. A1. Flower frontal view; B1. Flower ¾ view; A2. Flower frontal view; B2. Flower ¾ view. Photos by M.F. Monteros (A1, B1); Ron Parsons (B1, B2).

#### Paratype.

Ecuador • Imbabura: Reserva Río Manduriacu, 19 February 2024 (Coordinates omitted for conservation purposes; detailed data in the herbarium specimen), Marco F. Monteros, MFM312 (Paratype: QCNE!).

#### Eponymy.

The specific epithet is a noun in the genitive case, honoring Hildegarden Toeppfer de Kohn (1912–2006), a Czech refugee who settled in Ecuador in 1945. A devoted admirer of nature, particularly orchids, she inspired in her children and grandchildren a deep appreciation for the natural world. This legacy ultimately led them to establish the Río Manduriacu Reserve, the site where this species was discovered.

#### Phenology and flower variations.

Flowering individuals of *Porroglossum hildeae* have been observed in January, March, and November. During these periods, four plants were recorded in bloom, while six others remained non-flowering. The flowers exhibit variation in size and in the coloration of the veins on the dorsal and lateral sepals, ranging from dark brown, particularly on the adaxial surface of the apical sepal, to complete absence.

#### Distribution, habitat, and ecology.

**Figure 4.**
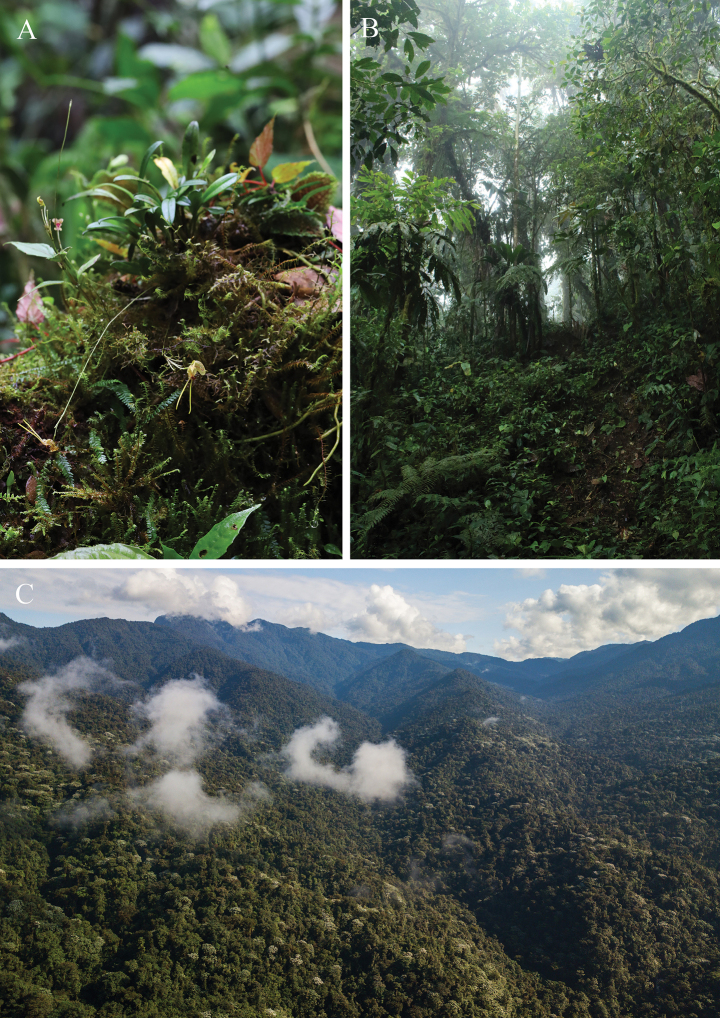
A. *Porroglossum hildeae* M.F.Monteros, E.Restrepo & Baquero growing in situ as an epiphyte on mossy branches in the Río Manduriacu Reserve; B. Cloud forest habitat of *P. hildeae*; C. Aerial view of the Río Manduriacu Reserve and landscape of the Cordillera del Toisán. Photos by M.F. Monteros (A, B); Jaime Culebras (C).

*Porroglossum hildeae* is so far restricted to two nearby locations within the Río Manduriacu Reserve in northwestern Ecuador, where it grows as an epiphyte at 1,600 m a.s.l. The habitat of the new species corresponds to a lower montane forest of the western Andes (BSN04), which is a humid ecosystem with high biodiversity located between 1,000 and 2,000 m a.s.l., under conditions of high cloud cover, and annual rainfall exceeding 2,000 mm (Fig. 4). It has a high coverage of medium and large trees, with abundant presence of epiphytes, mosses, tree ferns, and lianas (Ministerio de Ambiente de Ecuador 2013). It is a habitat for numerous endemic and threatened species, particularly orchids, birds, and amphibians, and plays a key role in water regulation in the Guayllabamba Basin in northwestern Ecuador (Roy et al. 2018; Baquero and Galarza-Verkovitch 2019; Guayasamin et al. 2019; Brito et al. 2020; Reyes-Puig et al. 2020; Monteros et al. 2022; Monteros et al. 2023). The newly described taxon grows sympatrically with other Pleurothallidinae species such as *Lepanthes kuijtii* Luer & Hirtz, *L. magnifica* Luer, *L. hexapus* Luer & R. Escobar, *Pleurothallis cauda-phocae* Luer & Hirtz, *Masdevallia nidifica* Rchb.f., *M. purocafeana* M.F. Monteros & Baquero, *Dracula erythrocodon* Luer & Dalström, and *Scaphosepalum dodsoni* Luer.

##### ﻿Preliminary conservation status

**Figure 5.**
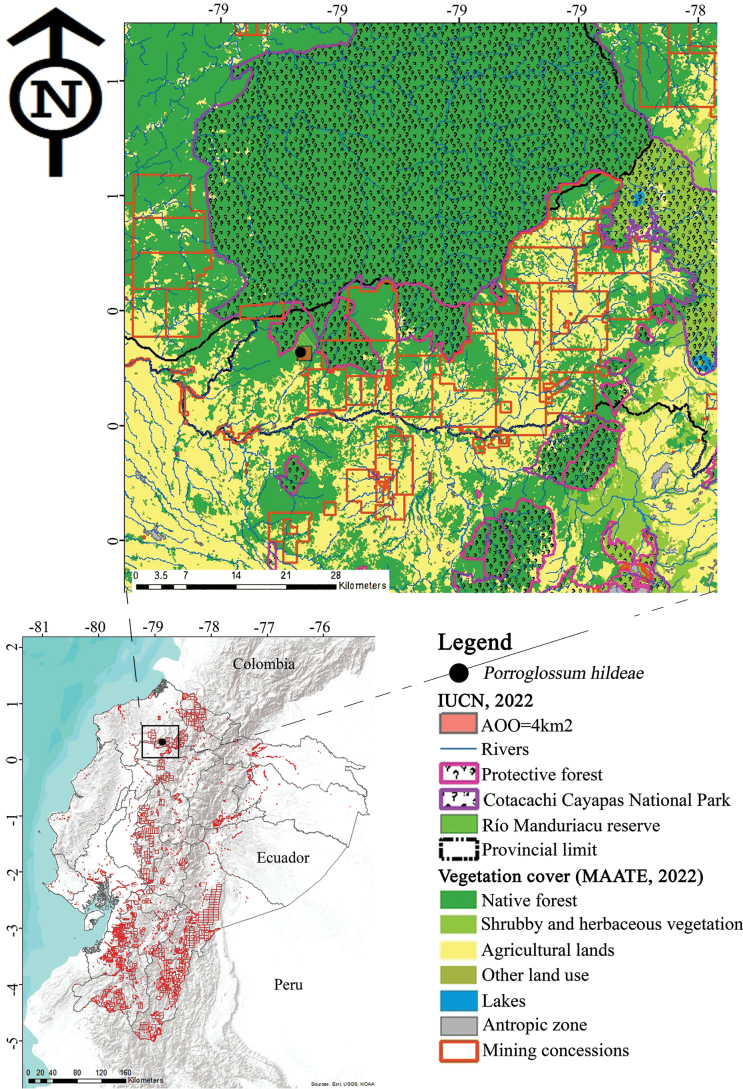
Distribution Map of *Porroglossum hildeae* M.F.Monteros, E.Restrepo & Baquero A. General view; B. conservation, land use, and Area of Occupancy AOO Map created by M. F. Monteros.

The newly described taxon is restricted to a single locality in northwestern Ecuador, with an extremely limited AOO, estimated at 4 km^2^, and is subject to intense and ongoing pressures, including deforestation and mining activities (Roy et al. 2018). The expansion of extractive industries and agricultural frontiers in northwestern Ecuador, combined with degradation of habitat quality, increases the risk of extinction (Fig. 5). Given these factors, we recommend that *P. hildeae* be classified as Critically Endangered (CR B2ab(iii,v)) according to the IUCN Red List Categories and Criteria (IUCN 2022), due to its restricted distribution, ongoing habitat loss, overexploitation, and potential mining activities in the region.

##### ﻿Taxonomic comments

A group of species of *Porroglossum* are known for having a considerably wider dorsal sepal compared to the lateral sepals, including *P. actrix* Luer & R. Escobar, *P. amethystinum* (Rchb. f.) Garay, *P. nutibara* Luer & R. Escobar, *P. aureum* Luer, *P. rodrigoi* H.R. Sweet, *P. hoeijerii* Luer, *P. marniae* Luer, *P. raoorum* Baquero & Iturralde, and *P. josei*, which was known to have the widest dorsal sepal in the genus (Baquero and Iturralde 2017) (Fig. 3). Nevertheless, with the discovery of *P. hildeae*, another species with an extremely wide dorsal sepal is added to the genus. Although the main differences between both species were outlined in the diagnosis, a few more morphological differences are discussed here: the new species can be distinguished from *P. josei* by the considerably larger plants (up to 20.0 cm including the inflorescence vs. up to 10 cm), the longer leaves (reaching 9.0 cm in length vs. up to 4.5 cm long), the wider, reniform, 5.0–7.0 × 17.0–20.0 mm dorsal sepal (vs. transversely ovate, 8.0 × 17.0 mm), the longer tails of the dorsal and lateral sepals (6.0 mm and 19.0–20.0 mm long respectively vs. 1.5 mm and 14.0 mm long), the oblong-ovate petals with slightly clawed apex with a single acute angle below the middle (vs. petals with subclavate apex and both margins sharply angled on both sides below the middle), the lip blade obtrullate, attenuate, acute, shortly pubescent towards the apex, (vs. glabrous, widely obovate, broadly obtuse, acuminate) (Figs 1, 2, 3).

*Porroglossum hildeae* is restricted to the southern sector of the Cordillera del Toisán (Guayllabamba River Basin), while the most similar species, *P. josei*, occurs north of the range in the Mira River Basin. This geographic disjunction suggests allopatric isolation mediated by the Cordillera del Toisán as a biogeographic barrier. Similar patterns have been documented in *Pristimantis* frogs (Reyes-Puig et al 2022; Yánez-Muñoz et al. 2025) and in the genus *Teagueia* (Jost 2004), where watershed isolation and microvicariance drive lineage diversification. These comparisons highlight Andean orogeny and topographic heterogeneity as recurrent drivers of speciation in the Tropical Andes (Reyes-Puig et al 2022; Jost 2004; Yánez-Muñoz et al. 2024; Yánez-Muñoz et al. 2025). In this context, the potential isolation of *P. hildeae* and *P. josei* provides additional evidence of these processes, though integrative morphological, genetic, and ecological studies remain necessary to confirm their evolutionary histories and independence.

## Supplementary Material

XML Treatment for Porroglossum hildeae

## ﻿Additional information

### Use of AI

No use of AI was reported.

### Author ORCIDs

Marco F. Monteros https://orcid.org/0000-0002-6670-3687

Eugenio Restrepo https://orcid.org/0000-0002-7037-1670

Gabriel A. Iturralde https://orcid.org/0000-0003-2456-0929

Marco M. Jiménez https://orcid.org/0000-0002-9502-5651

Luis E. Baquero https://orcid.org/0000-0002-1444-5727

### Data availability

All of the data that support the findings of this study are available in the main text.
